# Complete chloroplast genome of glyphosate resistant *Sonchus oleraceus* L. from Australia, with notes on the small single copy (SSC) region orientation

**DOI:** 10.1080/23802359.2018.1450682

**Published:** 2018-03-14

**Authors:** James P. Hereward, Jeff A. Werth, David F. Thornby, Michelle Keenan, Bhagirath Singh Chauhan, Gimme H. Walter

**Affiliations:** a School of Biological Sciences, The University of Queensland, Brisbane, QLD, Australia;; b Queensland Department of Agriculture and Fisheries, Leslie Research Centre, Toowoomba, QLD, Australia;; c Innokas Intellectual Services, Coomera, QLD, Australia;; d The Centre for Crop Science, Queensland Alliance for Agriculture and Food Innovation (QAAFI), The University of Queensland, Gatton, QLD, Australia

**Keywords:** *Sonchus oleraceus*, sowthistle, glyphosate resistance, weed

## Abstract

*Sonchus oleraceus*, common sowthistle, is an asteraceous weed in Australian agricultural systems and has recently developed resistance to glyphosate. We present the complete chloroplast sequence of *S. oleracueus* reconstructed from Illumina whole genome shotgun sequencing. This is the first complete chloroplast genome available for the genus *Sonchus*. The complete chloroplast sequence is 151,808 bp long. A Bayesian phylogeny of the chloroplast coding regions of the tribe Cichorieae (Asteraceae) is presented. The *S. oleraceus* chloroplast genome is deposited at GenBank under accession number MG878405.

*Sonchus oleraceus* (L.) (Asteraceae), common sowthistle, originated in Europe but is now cosmopolitan and found in almost every ice-free part of the globe (Gleason and Cronquist 1991; CABI 2018). This plant is edible, with antioxidant properties (Xia et al. 2011), but is also an agricultural weed. Australian populations with glyphosate resistance were detected in 2014 (Cook et al. 2014).

We assembled the complete chloroplast of *S. oleraceus* from a glyphosate resistant sample from the Liverpool plains region of New South Wales, Australia. A voucher is held by JPH at The University of Queensland. DNA was extracted from leaf using CTAB followed by DIY spin column purification (Ridley et al. 2016). Genomic sequencing libraries were constructed and a full lane of PE150 Illumina sequencing conducted by Novogene (Beijing, China). The chloroplast sequence was assembled in Geneious v11.1.3 (http://www.geneious.com, Kearse et al. 2012) by mapping to the *Lactuca sativa* (lettuce) complete chloroplast sequence (AP007232). This was followed by *de-novo* assembly of the matching reads and extension of the contigs using iterative read-mapping. Contigs were then ordered and joined by alignment to the reference. The final consensus sequence was checked manually to ensure correct mapping distances across the assembly. Annotations were based on alignment with lettuce.

All complete chloroplast sequences for the tribe Cichorieae were downloaded from GenBank. The complete chloroplasts were aligned using MAFFT (Katoh and Standley 2013). The GTR + I + G model of nucleotide substitution was found to be the most likely by jmodeltest2 (Darriba et al. 2012). A Bayesian phylogenetic tree was produced using Mr. Bayes (Huelsenbeck and Ronquist 2001) with *Helianthus annuus* as the outgroup (Figure 1).

**Figure 1.**
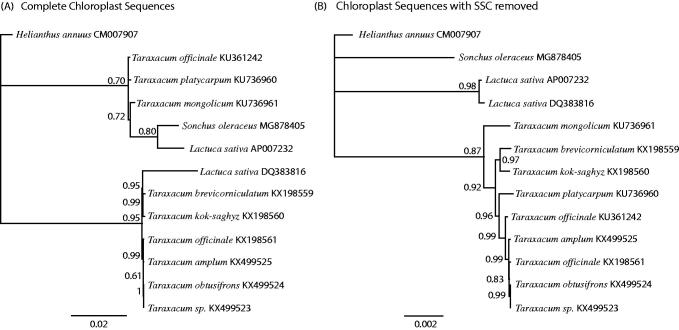
Phylogenetic tree produced using Bayesian estimation (Mr. Bayes) of complete chloroplast genomes from tribe Cichorieae (Asteraceae) (A), and chloroplast genomes with the Small Single Copy (SSC) region removed (B). The SSC is presented in both orientations in the same species on GenBank, and its removal produced a phylogeny much more congruent with taxonomy. *Helianthus annuus* was used as the outgroup, node labels indicate the posterior probability after 1 × 10^6^ iterations.

Chloroplasts typically have a quadripartite configuration with two inverted repeats (IRs), a Small Single Copy (SSC) region and a Large Single Copy (LSC) region. *Sonchus oleraceus* is no exception. Members of tribe Cichorieae on GenBank have the SSC presented in two orientations. We were unable to confirm one direction or the other for *S. oleraceus* by read mapping. The SSC can be found in both configurations within the same individual plant (Palmer 1983). Recently, this has been overlooked and the SSC has been reported as an inversion hotspot and its orientation used to make phylogenetic inferences as pointed out by (Walker et al. 2015). The representatives of Cichorieae on GenBank either have both configurations legitimately present (as per Palmer 1983) or there has been a mistake in assembly. The nature of the inverted repeats means that reads longer than and including the IRs (approx. 30kb) would be required to determine the exact orientation.

The tree based on the complete chloroplasts was misleading (Figure 1(A)). We produced a second tree with the SSC deleted (Figure 1(B)), which was more consistent with the taxonomy of these plants. These results demonstrate that care has to be taken when producing phylogenies based on complete chloroplast sequences. Chloroplast capture has been relatively common within subtribe Lactucinae (Cichorieae), (Wang et al. 2013) highlighting that it is not sensible to rely solely on chloroplasts for a phylogeny, even if they are complete ones.
